# Impaired Retrograde Transport Due to Lack of TBC1D5 Contributes to the Trafficking Defect of Lysosomal Cathepsins in Ischemic/Hypoxic Cardiomyocytes

**DOI:** 10.3389/fcvm.2021.796254

**Published:** 2021-12-23

**Authors:** Lin Cui, Qiong Zhang, Yao Huang, Lei Yang, Junhui Zhang, Xupin Jiang, Jiezhi Jia, Yanling Lv, Dongxia Zhang, Yuesheng Huang

**Affiliations:** ^1^Institute of Burn Research, State Key Laboratory of Trauma, Burns and Combined Injury, Southwest Hospital, Army Medical University (Third Military Medical University), Chongqing, China; ^2^Department of Endocrinology, Southwest Hospital, Army Medical University (Third Military Medical University), Chongqing, China; ^3^Department of Plastic Surgery, Southwest Hospital, Army Medical University (Third Military Medical University), Chongqing, China; ^4^Department of Wound Repair and Institute of Wound Repair, Shenzhen People's Hospital (The Second Clinical Medical College, Jinan University; The First Affiliated Hospital, Southern University of Science and Technology), Shenzhen, China

**Keywords:** lysosome, CI-MPR, retromer, TBC1D5, cardiomyocyte, ischemia/hypoxia

## Abstract

Lysosomal dysfunction has been found in many pathological conditions, and methods to improve lysosomal function have been reported to be protective against infarcted hearts. However, the mechanisms underlying lysosomal dysfunction caused by ischemic injury are far less well-established. The retromer complex is implicated in the trafficking of cation-independent mannose 6-phosphate receptor (CI-MPR), which is an important protein tag for the proper transport of lysosomal contents and therefore is important for the maintenance of lysosomal function. In this study, we found that the function of retrograde transport in cardiomyocytes was impaired with ischemia/hypoxia (I/H) treatment, which resulted in a decrease in CI-MPR and an abnormal distribution of lysosomal cathepsins. I/H treatment caused a reduction in TBC1D5 and a blockade of the Rab7 membrane cycle, which impeded retromer binding to microtubules and motor proteins, resulting in an impairment of retrograde transport and a decrease in CI-MPR. We also established that TBC1D5 was an important regulator of the distribution of lysosomal cathepsins. Our findings shed light on the regulatory role of retromer in ischemic injury and uncover the regulatory mechanism of TBC1D5 over retromer.

## Introduction

Cardiac ischemic/hypoxic injuries exist in many pathological conditions, including myocardial infarction, severe burns (1) and traumatic hemorrhage (2, 3), but the underlying mechanisms are still under mist. Lysosomes were initially discovered to be the primary digestive compartments by Christian de Duve in 1955. However, recent discoveries have uncovered their critical role as signal transduction platforms, regulators of cellular energy metabolism and cell death control. These functions of lysosomes interactively and cooperatively regulate cellular homeostasis (4–6). Lysosomal dysfunction, represented by the decreased degradation capacities of lysosomal hydrolases, is found in various pathological conditions, including lysosomal storage disorders (7, 8), neurodegenerative diseases (9–11) and cardiac ischemic injuries (12, 13). Therapeutic strategies targeting improving the activities of lysosomal hydrolases have been reported to ameliorate pathological damage (9, 12, 13).

Cation-independent mannose 6-phosphate receptor (CI-MPR) and cation-dependent mannose 6-phosphate receptor (CD-MPR) are two crucial receptors responsible for the proper trafficking of lysosomal proteins and the normal biogenesis of lysosomes. Lysosomal proteins transported by either receptor are distinct but largely overlapping. Evidence from *in vitro* binding assays has revealed that CI-MPR has a higher affinity and a broader spectrum for lysosomal proteins than CD-MPR (14). *In vivo* studies show that combined MPR deficiency causes pronounced mistargeting of lysosomal proteins with decreased activities of lysosomal enzymes in solid organs and elevated activities in the serum and accumulation of mannose 6-phosphorylated glycoproteins. Deficiency in either receptor causes a milder phenotype under physiological conditions, but CI-MPR deficiency is much more severe (15–19).

After transporting lysosomal proteins to endosomes from the Golgin apparatus, MPRs are recycled back to avoid being digested in lysosomes. The trafficking of CI-MPR between the Golgin apparatus and endosomes was previously considered to be regulated by retromer, a multiprotein complex, and disruption of retrograde transport causes rapid degradation and mislocalization of CI-MPR (20–22). However, recently, some studies have reported that the retrograde transport of CI-MPR is retromer-independent under normal conditions (23, 24). Studies regarding the function of the myocardial retromer have not been reported.

The classic retromer complex comprises a VPS26-VPS29-VPS35 heterotrimer mainly responsible for cargo recognition and referred to as the cargo selective complex (CSC) and a heterodimer of SNX1/2 and SNX5/6 that is implicated in membrane recruitment and the formation of recycling tubules (25, 26). The retromer complex is recruited to endosomal membranes and recognizes its substrates with the help of Rab7 GTPase and SNX3 (27–33). The local enrichment of the retromer-substrate complex on endosomal membranes promotes the remodeling of the endosomal membranes to gradually form the tubule structure before detaching from the endosomal membranes (34). Although the mechanisms regarding the assembly and recruitment of retromer CSCs have been intensively investigated, the machinery underlying the later processes, including membrane uncoating and fission, is far less well-understood. TBC1D5, the relatively specific GTPase activating protein (GAP) for Rab7, promotes Rab7 inactivation and its dissociation from late endosomal membranes, which should theoretically negatively regulate the function of the retromer CSC (35–37). Although a portion of studies have supported this idea, a few studies suggest that TBC1D5 deletion disrupts retromer-mediated receptor recycling and alters the distribution of CI-MPR, indicative of its indispensable role in retrograde transport (38–40). TBC1D5 can form an astoichiometric complex with CSCs through a binary interaction involving both VPS35 and VPS29, which might suggest an important regulatory role for the retromer coat (38).

The regulatory role of TBC1D5 in the retromer complex is still controversial. Whether TBC1D5 functions in ischemic cardiomyocytes has not been investigated. In the present study, we found that ischemia induced significant loss of TBC1D5, which caused the blockade of retrograde transport and a decrease in CI-MPR. TBC1D5 was indispensable for the regulation of the retromer complex by affecting its connection with microtubules. Restoration of TBC1D5 also alleviated the trafficking deficit of lysosomal cathepsins caused by I/H injury, which might suggest a new therapeutic target for ischemic injury.

## Materials and Methods

### Reagents and Antibodies

Bafilomycin A1 (Baf A1, 20 μM) was purchased from Selleck (USA), and the classical autophagy inhibitor 3-methyladenine (3-MA, 1 mM) was purchased from Sigma-Aldrich (USA). Protein A/G-Sepharose (Santa Cruz, USA) was used in the immunoprecipitation assays. The antibodies used in this study included rabbit anti-MPR (cation independent) antibody (Abcam, USA), rabbit anti-α-Tubulin antibody (Proteintech, USA), rabbit anti-cathepsin B antibody (Santa Cruz, USA), mouse anti-cathepsin D antibody (Santa Cruz, USA), rabbit anti-Lamp1 antibody (Abcam, USA), rat anti-Lamp1 antibody (Abcam, USA), mouse anti-Golgin 160 antibody (Santa Cruz, USA), mouse anti-EEA1 antibody (Santa Cruz, USA), mouse anti-GLUT4 antibody (Santa Cruz, USA), mouse anti-TBC1D5 antibody (Santa Cruz, USA), mouse anti-VPS35 antibody (Santa Cruz, USA), mouse anti-VPS29 antibody (Santa Cruz, USA), mouse anti-SNX1 antibody (Santa Cruz, USA), mouse anti-SNX2 antibody (Santa Cruz, USA), mouse anti-SNX5 antibody (Santa Cruz, USA), mouse anti-SNX6 antibody (Santa Cruz, USA), mouse anti-Rab7 antibody (Abcam, USA), HRP-conjugated mouse anti-Actin antibody (Proteintech, USA), mouse anti-α-Tubulin antibody (Proteintech, USA), rabbit anti-ATG5 antibody (Cell Signaling Technology, USA) and rabbit anti-Dynactin p150glued antibody (Cell Signaling Technology, USA).

### Cell Culture and Ischemia/Hypoxia Treatment

Neonatal C57BL/6J mice (1–3 days) were obtained from the Animal Center of the Army Medical University (Third Military Medical University), and all animal experiments were approved by the Animal Experiment Ethics Committee and performed according to the Guide for the Care and Use of Laboratory Animals published by the US National Institutes of Health (NIH Publication, 8th Edition, 2011). Neonatal mouse cardiomyocytes were cultured according to protocols published previously (1). Briefly, the neonatal mouse hearts were separated quickly, trimmed and washed completely before being cut into tiny pieces. The tissue fragments were digested by collagenase type II and trypsin to isolate cells. The cell suspension was cultured in the cell incubator for 2 h to allow the fibroblasts to adhere first and separate them from cardiomyocytes. Then, the cells were plated and used for the following experiments. Cardiomyocytes were changed to serum- and glucose-free medium and incubated in a hypoxic CO_2_ incubator (Thermo Scientific, USA) filled with 94% N_2_, 5% CO_2_ and 1% O_2_ in the I/H group. Reagents were added 1 h before the treatment.

### Adenovirus Infection

Scarlet-Rab7 adenovirus was constructed by OBiO Technology (Shanghai, China). Cardiomyocytes were cultured on confocal dishes for 24 h before infection with Scarlet-Rab7 adenovirus according to the instructions. After 36–48 h of infection, cardiomyocytes were subjected to the corresponding treatment, and fluorescence recovery after photobleaching (FRAP) assays were performed. Adenoviruses carrying full-length TBC1D5 were purchased from OBiO Technology (Shanghai, China), and infection experiments were carried out according to the manufacturers' instructions. Western blotting was used to detect the infection efficiencies, and cells were subjected to the following experiments after infection for 36–72 h.

### Gene Silencing With siRNAs

ATG5 siRNAs, VPS29 siRNAs and TBC1D5 siRNAs were constructed by GenePharma (Shanghai, China), and siRNAs were transfected into cardiomyocytes with Lipofectamine 2000 (Invitrogen, USA) after culturing for 24–36 h according to the instructions. Transfection efficiencies were examined by western blot, and cells were used in subsequent experiments after transfection for 36–72 h.

### Western Blot Assay

Cardiomyocytes were lysed in RIPA buffer containing protease inhibitor tablets, and the protein concentrations were examined with Quick Start^TM^ Bradford 1x dye reagent (BioRad, USA) before being separated on an SDS-PAGE gel (Bio-Rad, USA). Then, the proteins were transferred to PVDF membranes (Millipore, USA), blocked with 5% skim milk and incubated with the corresponding primary antibodies and secondary antibodies. The protein bands were visualized with avidin biotinylated horseradish peroxidase with an enhanced chemiluminescence detection kit (GE Healthcare, USA).

### Immunofluorescence Assay and Confocal Microscope

After the treatment, cardiomyocytes cultured on glass coverslips were fixed with 4% paraformaldehyde for 20 min and blocked with 5% bovine serum albumin for 1–3 h at room temperature. Then, the cells were incubated with specific primary antibodies at 4°C overnight and the corresponding secondary antibodies at 37°C for 1 h. 4,6-diamino-2-phenyl indole (DAPI) was used to stain the nuclei. Images were captured with a confocal microscope (TCS-NT, Leica, Germany).

For colocalization analysis, the integrated optical density (IOD) of the yellow area and the area of the corresponding cell were analyzed by the Image Pro Plus 6.0 software (Media Cybernetics, Silver Spring, MD, USA), respectively. Then the ratio of the IOD to the area was calculated to represent the colocalization value. The experiments were repeated three times independently, and a mean of 3 means was calculated.

### Fluorescence Recovery After Photobleaching

Cardiomyocytes were cultured on confocal dishes with glass bottoms and infected with Scarlet-Rab7 adenovirus before the FRAP experiments. FRAP experiments were performed using a confocal microscope (TCS-NT, Leica, Germany). Two prebleaching images were taken to record the initial fluorescence intensity, and the selected area was bleached at maximal intensity 4 times using a 568 nm laser. Then, fluorescence images were captured every 1.318 s for ~2 min.

### Immunoprecipitation

To determine the binding of the retromer to the cytoskeleton and the motor protein, cardiomyocytes were lysed in RIPA buffer containing protease inhibitor tablets, and the protein lysate was then incubated with VPS35 antibody (Santa Cruz) for 6 h at 4°C before the complex was precipitated with protein A/G-Sepharose (Santa Cruz) overnight at 4°C. The precipitate was washed 3 times with ice-cold PBS and separated by SDS/PAGE using western blotting.

### Statistical Analysis

Quantification was performed in three independent experiments, and statistical analysis was performed using SPSS 13.0 software. The significance of differences was evaluated by unpaired Student's *t*-test or one-way analysis of variance (ANOVA) followed by *post hoc* tests. A *P*-value < 0.05 was considered statistically significant.

## Results

### I/H Treatment Impairs the Trafficking of Lysosomal Cathepsins in Cardiomyocytes

**Figure 1:**
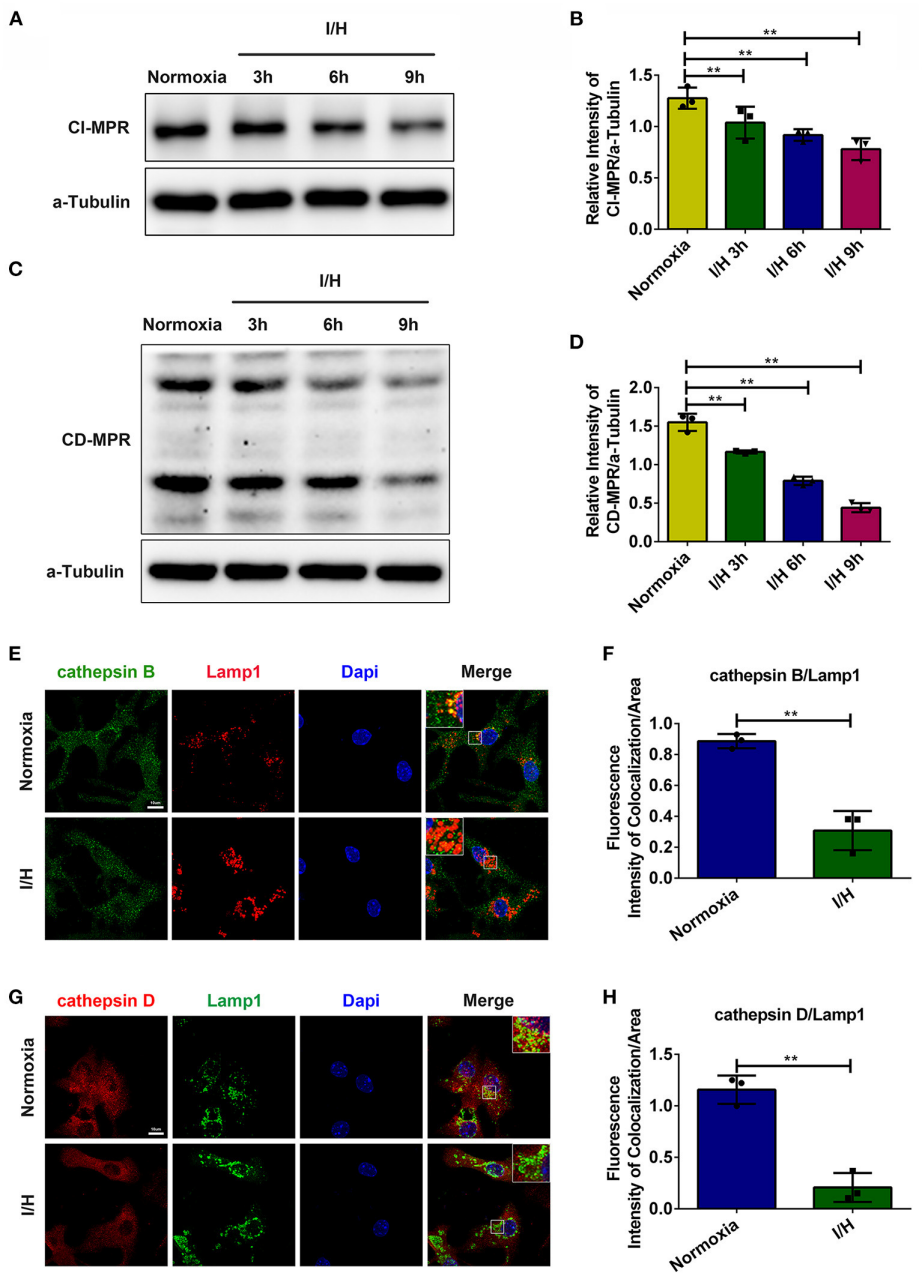
I/H treatment disrupted the trafficking of lysosomal cathepsins. **(A)** Western blotting was performed to detect the protein levels of CI-MPR after I/H treatment for different periods. **(B)** Quantitative analysis of immunoblots in **(A)**. **(C)** Western blotting was performed to detect the protein levels of CD-MPR after I/H treatment for different periods. **(D)** Quantitative analysis of immunoblots in **(C)**. **(E,F)** Representative confocal images **(E)** and quantitative analysis **(F)** of the fluorescence intensity of colocalization per area between cathepsin B and Lamp1 after I/H treatment for 9 h. **(G,H)** Representative confocal images **(G)** and quantitative analysis **(H)** of the fluorescence intensity of colocalization between cathepsin D and Lamp1 after I/H treatment for 9 h. All scale bars = 10 μm, all error bars = SD, and **P < 0.01 of the respective condition compared to the control group.

Previous studies have reported that myocardial infarction causes a decrease in the activities of lysosomal hydrolases accompanied by elevated activities in the serum, which contributes to heart dysfunction (12, 13). The redistribution of lysosomal hydrolases resembles the phenotype of MPR-deficient mice (13, 15). Consequently, we suspected that lysosomal dysfunction caused by ischemia injury might correlate with the blockade of lysosomal cathepsin trafficking due to the lack of MPRs. We first performed immunostaining and colocalization analysis of cathepsins with lysosomal membrane-associated protein 1 (Lamp1). As shown in Figures 1E–H, I/H treatment significantly decreased the colocalization between cathepsins and Lamp1, especially cathepsin D. Meanwhile, we examined the protein levels of MPRs. In accordance, the protein level of CI-MPR presented a time-dependent decrease (Figures 1A,B), and CD-MPR showed similar changes (Figures 1C,D). These results suggested that I/H treatment impeded the trafficking of cathepsin B and cathepsin D, which probably contributed to the dysfunction of lysosomes.

### The Redistribution of CI-MPR Caused by I/H Treatment Is Retromer-Dependent

**Figure 2:**
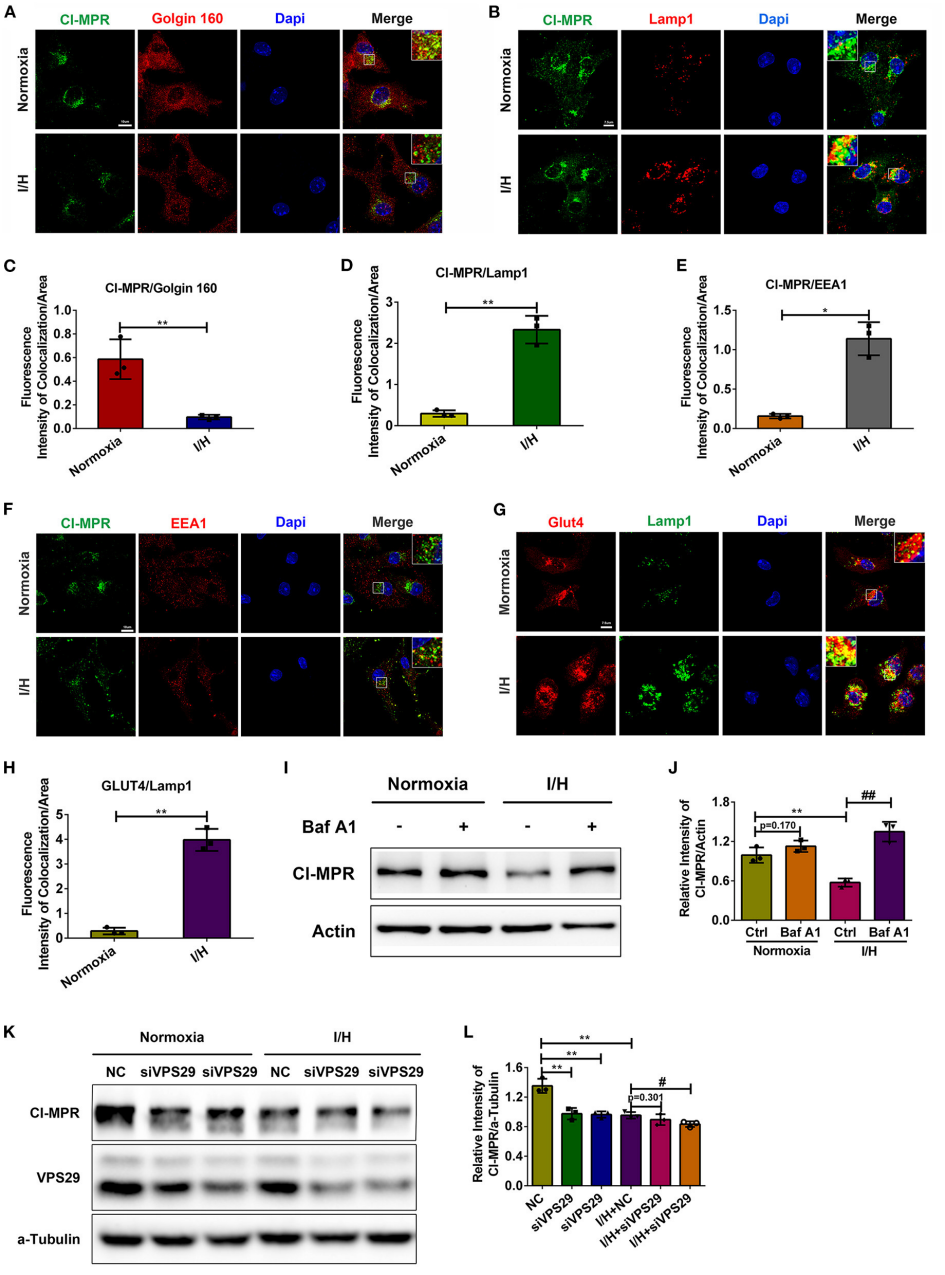
I/H treatment caused impairment of retromer function and abnormal distribution of CI-MPR. **(A,C)** Immunofluorescent colocalization staining was performed to detect the distribution of CI-MPR in the Golgin apparatus with I/H treatment **(A)**, and the quantitative analysis is shown in **(C)**. Scale bar, 10 μm. **(B,D)** Representative confocal images **(B)** and quantitative analysis **(D)** of the fluorescence intensity of colocalization per area between CI-MPR and Lamp1 after I/H treatment. Scale bar, 7.5 μm. **(E,F)** Representative confocal images **(F)** and quantitative analysis **(E)** of the fluorescence intensity of colocalization per area between CI-MPR and EEA1 with I/H treatment. Scale bar, 10 μm. **(G,H)** The lysosomal distribution of Glut4 was also detected with the immunofluorescent staining **(G)**, and the quantitative analysis of the fluorescence intensity of colocalization per area is shown in **(H)**. Scale bar, 7.5 μm. **(I)** Baf A1 (20 μM) was applied to inhibit the activities of lysosomal hydrolases, and the protein levels of CI-MPR were determined by western blotting. **(J)** Quantitative analysis of the immunoblots in **(I)**. **(K)** VPS29 siRNAs were transfected into cardiomyocytes to disturb the function of the retromer under both control and I/H conditions. Western blotting was used to detect the changes in CI-MPR. **(L)** The results of the quantitative analysis in **(K)**. All error bars = SD, **P < 0.01, *P < 0.05 of the respective condition compared to the control group and ^##^*P* < 0.01, ^#^*P* < 0.05 of the respective condition compared to the I/H group.

Although MPRs are expressed in most cell types, CI-MPR is much more abundant in the cardiovascular system than CD-MPR (41). Moreover, deficiency in CI-MPR is more pronounced in the missorting of lysosomal proteins. Therefore, here, we paid attention to the mechanism of the decrease in CI-MPR. We first performed immunostaining and colocalization analysis of CI-MPR and markers of different organelles. As shown in Figures 2A–D, CI-MPR was concentrated primarily in the Golgin apparatus with minimal localization in Lamp1-positive late endosomes/lysosomes under physiological conditions, which is consistent with previous studies. However, I/H treatment caused a significant alteration of its distribution. CI-MPR promptly translocated to late endosomes/lysosomes with a remarkable reduction in the Golgin apparatus. The colocalization between CI-MPR and the early endosome marker EEA1 also increased with I/H stress (Figures 2E,F). Considering that lysosomes/late endosomes contain so many capable hydrolases, the retention could accelerate their degradation and cause a reduction in the protein content. To validate this hypothesis, we applied Baf A1 to inhibit the activities of lysosomal hydrolases. The result that the protein level of CI-MPR increased significantly in the I/H plus Baf A1 group, comparable with the control plus Baf A1 group, supported this assumption (Figures 2I,J). We also performed immunostaining of another substrate of retrograde transport, glucose transporter 4 (GLUT4) (42, 43). In accordance, I/H treatment similarly induced robust localization in Lamp1-positive late endosomes/lysosomes compared with the control group (Figures 2G,H). Considering that I/H treatment induces the activation of autophagy and the accompanying acceleration of anterograde transport, lysosomal retention of the above substrates could result from impaired retrograde transport. Therefore, we next applied 3-methyladenine (3-MA) and ATG5 siRNAs to inhibit autophagy and reduce anterograde transport. As expected, both 3-MA and ATG5 siRNAs reversed the downregulation of CI-MPR caused by I/H treatment (Supplementary Figure S1). The data above collectively supported that I/H treatment caused the impairment of retrograde transport in cardiomyocytes and therefore contributed to the degradation of CI-MPR in lysosomes.

Given that the retromer complex is the core component for retrograde transport, we next aimed to validate whether the function of the retromer regulates the protein content of CI-MPR in cardiomyocytes. We used siRNAs to downregulate the protein level of VPS29. Loss of VPS29 significantly decreased the protein level of CI-MPR under physiological conditions, suggesting that the regulation of CI-MPR in cardiomyocytes is retromer-dependent. The extent of the reduction in CI-MPR in the I/H group was much smaller, which indicated that the function of the retromer was damaged by I/H treatment and that depletion of VPS29 could not further exacerbate this condition (Figures 2K,L). These results further suggested that the decrease in CI-MPR caused by I/H stress is retromer-dependent in cardiomyocytes.

### The Retromer Complex Itself Remains Unchanged Upon I/H Stress

To further investigate the underlying mechanism accounting for the impaired function of the retromer, we first detected their protein contents. As shown in Supplementary Figures 2A–G, the protein levels of VPS35 and VPS29, two important components of the VPS trimer, and SNX1, SNX2, SNX5, and SNX6, components of the SNX dimer, presented no significant differences in response to I/H treatment. We next examined the colocalization between the retromer and Lamp1 to investigate the endosomal recruitment of the retromer complex. The colocalization between VPS35 and Lamp1 increased remarkably with I/H treatment, and the results of SNX6 showed similar changes (Supplementary Figures 2H–K), suggesting that the connection between the retromer and the late endosomes was increased with I/H treatments. The data above suggested that the protein contents and recruitment of the retromer complex did not account for the impairment of retrograde function with I/H treatment.

### Decrease in TBC1D5 Accounts for the Loss of CI-MPR With I/H Treatment

**Figure 3:**
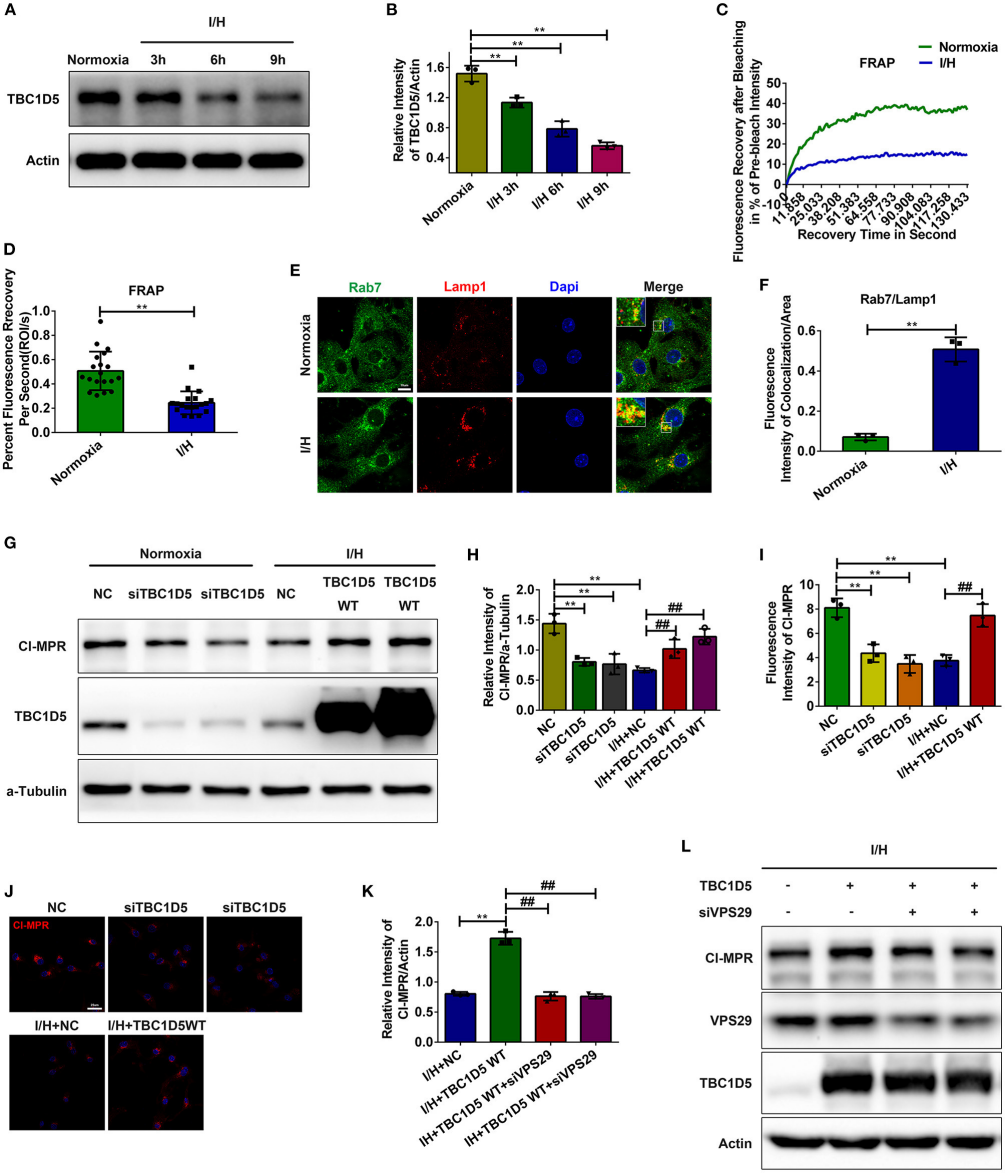
TBC1D5 regulates CI-MPR through retromer in cardiomyocytes. **(A,B)** The protein levels of TBC1D5 were determined by western blotting **(A)**, and the results of the quantitative analysis are shown in **(B)**. **(C,D)** Primary neonatal mouse cardiomyocytes were infected with Scarlet-tagged Rab7 adenovirus to perform FRAP assays and detect the membrane cycle of Rab7. The curve of the percent recovery of the prebleach intensity is shown in **(C)**, and the quantitative analysis of the recovery rate is shown in **(D)**. **(E,F)** Immunofluorescent colocalization staining was used to detect the distribution of Rab7 under both control and I/H conditions **(E)**, and the results of the quantitative analysis are shown in **(F)**. Scale bar, 10 μm. **(G)** TBC1D5 siRNAs and adenovirus carrying full-length TBC1D5 were used to decrease and increase its protein levels in control and I/H conditions, respectively. Western blotting was performed to determine the corresponding changes in CI-MPR. **(H)** Quantitative analysis of the immunoblots in **(G)**. **(I,J)** Representative images of CI-MPR with TBC1D5 siRNA and TBC1D5-overexpressing adenovirus in control and I/H conditions, respectively **(J)**, and the quantitative analysis of its immunofluorescent intensities **(I)**. Scale bar, 25 μm. **(K,L)** VPS29 siRNAs were transfected into cardiomyocytes to impair the function of the retromer with TBC1D5 overexpression under I/H conditions. The changes in CI-MPR were detected by western blotting **(L)**, and the results of the quantitative analysis are shown in **(K)**. All error bars = SD, **P < 0.01 vs. the control cells and ^##^*P* < 0.01 vs. the I/H group.

Increased colocalization between the retromer and late endosomes/lysosomes with I/H treatment suggested retarded dissociation with increased recruitment of the retromer complex. Therefore, we next examined the changes in Rab7 and its GAP TBC1D5, which promote the recruitment and dissociation of the retromer complex, respectively. We observed a time-dependent decrease in TBC1D5 induced by I/H treatment (Figures 3A,B), with Rab7 gradually redistributing to lysosomes/late endosomes compared to the even distribution in the cytoplasm under normal conditions (Figures 3E,F). Given that TBC1D5 accounts for proper inactivation and dissociation from the endosomes of Rab7, we next aimed to determine whether the shifted active state of Rab7 was accompanied by the loss of motility and membrane cycling. We performed fluorescence recovery after photobleaching (FRAP) assays of mScarlet-Rab7. Under normal conditions, the fluorescence of the mScarlet-Rab7 signal recovered rapidly after photobleaching, while the I/H group showed remarkably decreased overall recovery extent and retarded recovery rate (Figures 3C,D). These data suggested that I/H treatment caused the loss of TBC1D5 and a deficit in Rab7 membrane recycling.

The role of TBC1D5 in regulating the function of retromer is still controversial (37, 38). To investigate whether TBC1D5 deficiency induced by I/H treatment causes a decrease in CI-MPR, we used siRNAs to decrease the expression of TBC1D5 under normal conditions and adenovirus carrying full-length TBC1D5 to restore its protein content with I/H treatment. TBC1D5 knockdown in the control group resulted in a significant decrease in CI-MPR, while overexpression of TBC1D5 significantly rescued its loss with I/H treatment (Figures 3G,H). Immunofluorescence of CI-MPR presented similar results (Figures 3J,K). Moreover, the elevated protein content of CI-MPR with TBC1D5 overexpression was reversed by VPS29 knockdown in the I/H group (Figures 3I,L). These data suggested that TBC1D5 deficiency induced by I/H stress accounts for the decrease in CI-MPR through retromer-dependent pathways.

### TBC1D5 Prevents the Trafficking Deficit of Lysosomal Cathepsins

**Figure 4:**
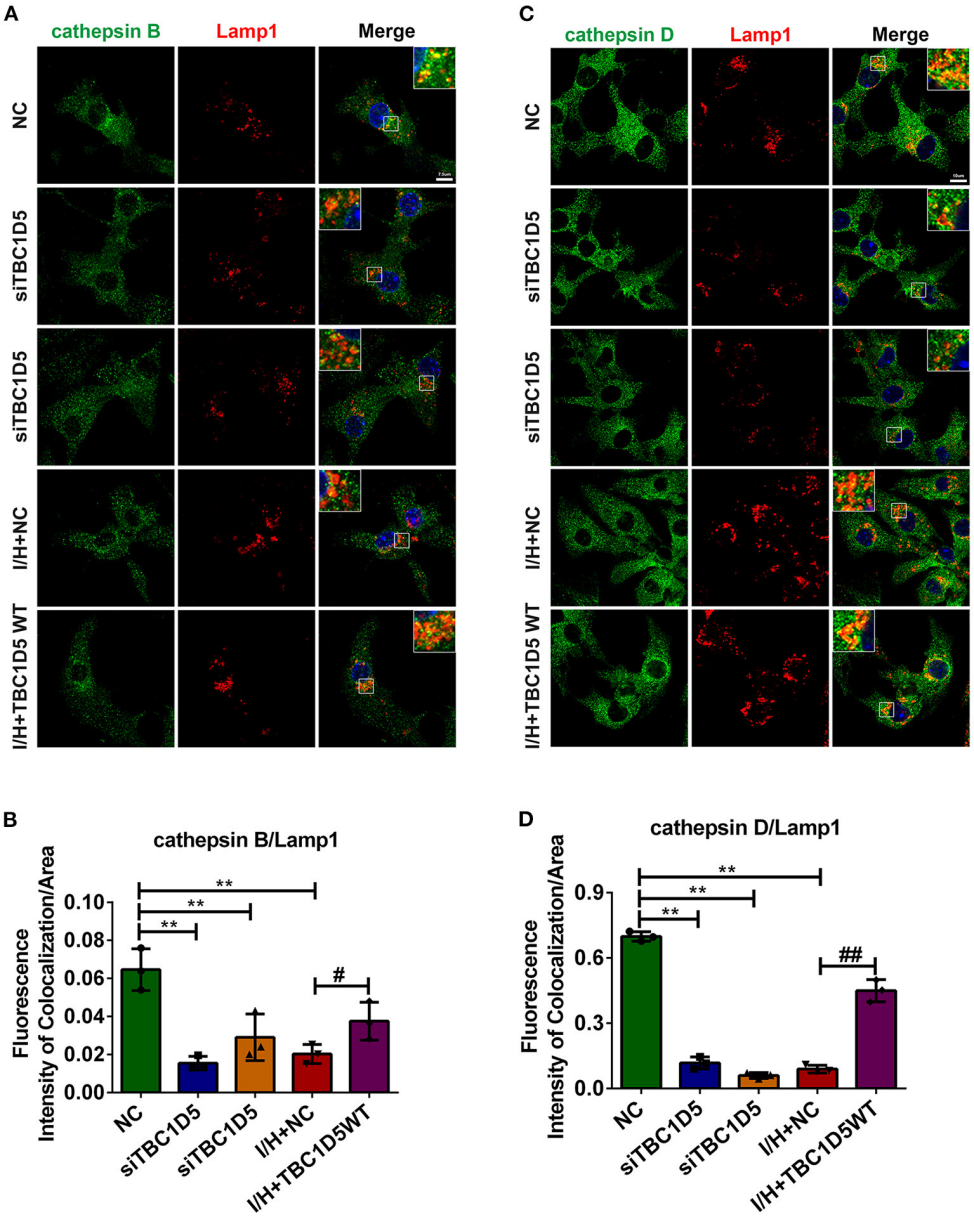
The trafficking of lysosomal cathepsins was regulated by TBC1D5 in cardiomyocytes. **(A,B)** TBC1D5 siRNAs were used to downregulate its protein level under control conditions, and adenovirus carrying full-length TBC1D5 was used to restore its protein content with I/H treatment. The trafficking of cathepsin B was determined by immunofluorescent colocalization staining **(A)**, and the quantitative analysis is shown in **(B)**. Scale bar, 7.5 μm. **(C)** Immunofluorescent colocalization staining of cathepsin D and Lamp1 was performed to assay its trafficking. Scale bar, 10 μm. **(D)** Quantitative analysis of the fluorescence intensity of colocalization per area between cathepsin D and Lamp1. All error bars = SD, **P < 0.01 vs. the control cells and ^##^*P* < 0.01, ^#^*P* < 0.05 vs. the I/H group.

CI-MPR is widely accepted as an important protein for the proper trafficking of lysosomal cathepsins, so we wondered whether the amount of CI-MPR regulated by TBC1D5 could affect the trafficking of lysosomal cathepsins. As expected, TBC1D5 knockdown significantly impeded while TBC1D5 overexpression remarkably improved the trafficking of cathepsin D (Figures 4C,D). The trafficking of cathepsin B showed similar changes to a smaller extent (Figures 4A,B). The proper trafficking of lysosomal cathepsins could partially prevent their abnormal secretion and mislocalization, which might alleviate the cytotoxicity of cardiomyocytes.

### TBC1D5 Regulates the Retrograde Transport by Affecting the Association Between the Retromer Complex and Microtubules

**Figure 5:**
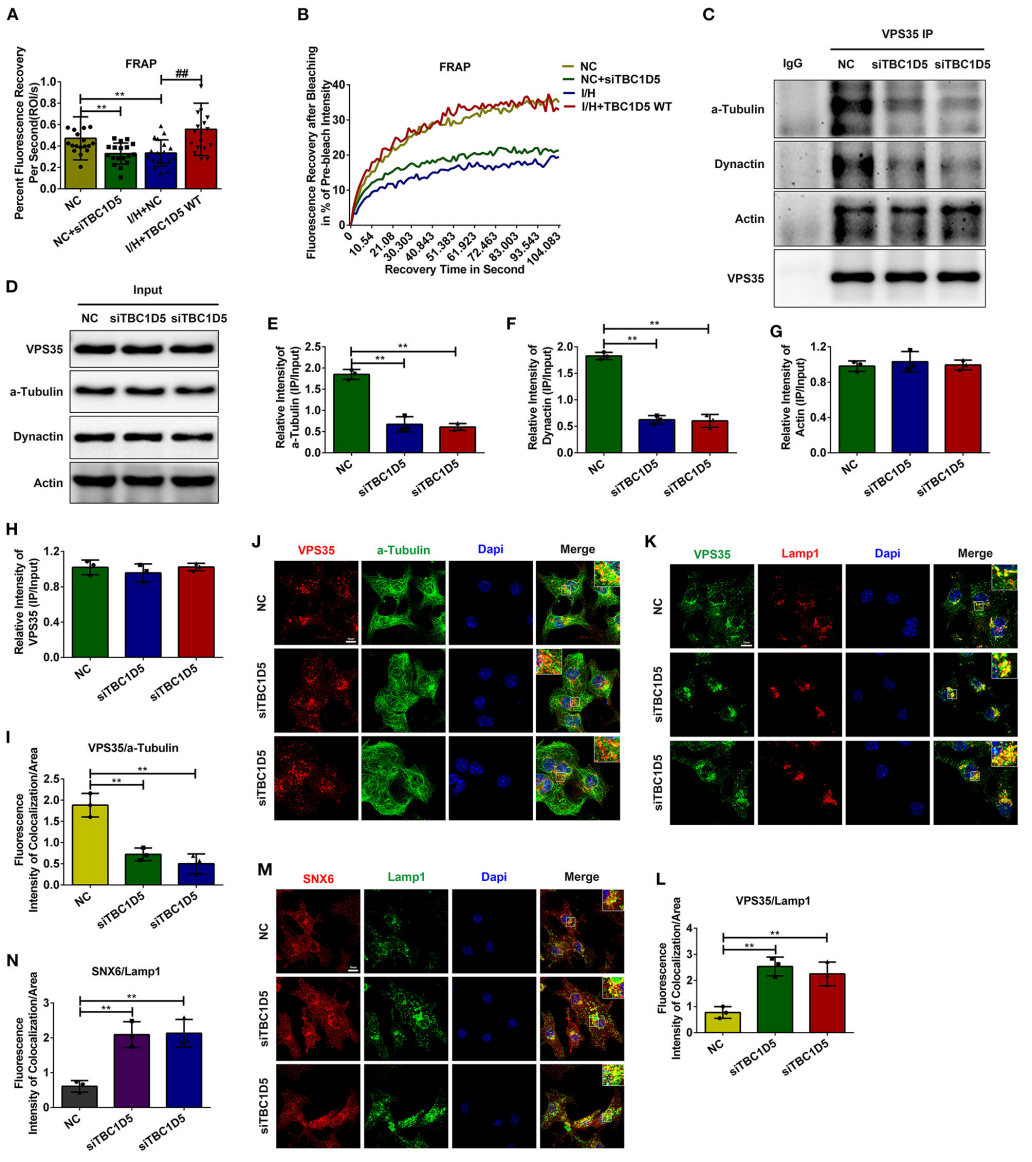
TBC1D5 affected the connection of the retromer with the microtubule. **(A,B)** FRAP assays were performed to detect the membrane cycle of Scarlet-Rab7 with TBC1D5 siRNA under control conditions and TBC1D5 overexpression under I/H conditions. The curve of the percent recovery of Rab7 is shown in **(B)**, and the quantitative analysis of its recovery rate is shown in **(A)**. **(C,D)** Cardiomyocytes were transfected with TBC1D5 siRNAs under normal conditions. After lysis with RIPA buffer, the protein lysates were recovered by IP with VPS35 antibody. The immunoprecipitated proteins were analyzed by western blotting **(C)**, and the overall levels of the target proteins between different groups were also detected **(D)**. **(E–H)** Quantitative analysis of α-tubulin **(E)**, dynactin p150^glued^ **(F)**, actin **(G)**, and VPS35 **(H)** in **(C)**. **(I,J)** The colocalization between the retromer and tubulin with TBC1D5 siRNAs was determined by the immunofluorescent staining **(J)** and quantitative analysis **(I)**. **(K)** The connection of the retromer with the endosomal membrane was analyzed by the colocalization between VPS35 and Lamp1 using immunofluorescent staining. **(L)** Quantitative analysis of the immunofluorescent colocalization staining in **(K)**. **(M)** The immunofluorescent colocalization staining of SNX6 and Lamp1 with TBC1D5 siRNA treatment. **(N)** Quantitative analysis in **(M)**. All scale bars, 10 μm. All error bars = SD, **P < 0.01 of the respective condition compared to the control group and ^##^*P* < 0.01 of the respective condition compared to the I/H group.

The data above suggested that TBC1D5 was an important regulator of CI-MPR and the trafficking of lysosomal cathepsins through retromer-dependent pathways. Therefore, we next aimed to determine how TBC1D5 affected the function of the retromer complex. As observed in Supplementary Figure 3, genetic knockdown or overexpression of TBC1D5 did not change the protein contents of the retromer complex. TBC1D5 has been widely reported to regulate the function of retromer by affecting the membrane cycle of Rab7 (35–40). Therefore, we next performed FRAP assays of Rab7 with TBC1D5 siRNA and adenovirus. As observed in Figures 5A,B, loss of TBC1D5 under normal conditions caused a significantly decelerated rate and decreased extent of Rab7 membrane recycling, while overexpression of TBC1D5 remarkably reversed this reduction caused by I/H treatment. A study using live cell imaging found that retromer tubules usually emanate from endosomal foci, where Rab7 is most concentrated, and the content of Rab7 decreases when tubules detach from endosomes (28). Therefore, we wondered whether the decelerated membrane detachment of Rab7 caused by TBC1D5 deficiency affected the dissociation of the retromer. We observed that lysosomes in the TBC1D5 siRNA group were much larger than the lysosomes in the normal group and that more retromer was localized on late endosomes/lysosomes with TBC1D5 siRNAs (Figures 5K–N). The detachment of the retromer-cargo complex has been reported to require longitudinal force by the microtubule and its motor proteins, and inhibition of the dynein-dynactin complex causes either the enlargement of retromer-labeled endosomes with minimal tubules or the abnormal elongation of tubules that fail to be sustained and are quickly retracted (20, 44). Therefore, we next conducted IP assays and immunofluorescence colocalization analysis to examine the connection of the retromer with the cytoskeletal system. The association of VPS35 with α-tubulin remarkably decreased with TBC1D5 knockdown under normal conditions (Figures 5C–E,I,J). Furthermore, its connection to dynactin p150^glued^ also decreased in the TBC1D5 siRNA group, with no significant changes in the connection to actin (Figures 5C,D,F,G). The data above suggested that TBC1D5 deficiency disturbed the association of the retromer with the microtubule, which impeded the exit of the retromer.

## Discussion

Lysosomal dysfunction has previously been found in myocardial infarction with unknown mechanisms and methods to improve the function of lysosomes alleviate myocardial injury (13, 45). Here, we discovered that defects in retrograde transport caused a decrease in CI-MPR, which could contribute to the impairment of lysosomal cathepsin trafficking. We also identified a role for TBC1D5 in regulating the trafficking of lysosomal cathepsins through its effect on the retromer complex, and it could be a potent therapeutic target in improving lysosomal function in the ischemic heart.

MPRs have been widely accepted to be important adaptors for lysosomal components and CI-MPR is more pronounced for proper trafficking with higher affinity and broader spectrum than CD-MPR (14–19). Furthermore, the results from a developmental study show that CI-MPR is much more abundant than CD-MPR in the cardiovascular system (41). In this study, we found that CI-MPR gradually decreased, and the distribution of lysosomal cathepsins remarkably decreased with I/H treatment, which suggested a trafficking defect of lysosomal hydrolases and could explain the dysfunction of myocardial lysosomes. We also found that the distribution of a large portion of CI-MPR shifted from the Golgin apparatus to the endolysosomal system and that inhibition of the activities of lysosomal hydrolases or the induction of autophagy in the I/H group reversed the reduction in CI-MPR. Considering that autophagy induction could cause the activation of the endolysosomal system and promote the transport of CI-MPR from the Golgin apparatus to endosomes, these results suggested the inhibited retrograde transport of CI-MPR with I/H treatment. Although there are different opinions about the trafficking of CI-MPR (23, 24), our results supported that the protein level of CI-MPR was regulated by retromer in cardiomyocytes. These results were consistent with previous studies showing that inhibition of retromer causes a decrease in CI-MPR and lysosome dysfunction (21, 22). Under conditions of I/H injury, when cellular autophagy is robustly induced (46, 47) and the demand for retrograde transport is significantly upregulated, dysfunction of the retromer is probably destructive to cardiomyocytes.

To date, the inhibition of cellular retrograde transport in I/H conditions has not been investigated, and the underlying mechanisms remain unclear. We observed that the lysosomal distribution of the retromer was increased with I/H treatment and that Rab7 was hyperactivated with a remarkable deceleration of its motility due to a lack of TBC1D5. We demonstrated that both the protein level of CI-MPR and the membrane cycle of Rab7 were regulated by TBC1D5 in cardiomyocytes. Overexpression of TBC1D5 with I/H treatment restored the protein content of CI-MPR, which could decrease the abnormal secretion of lysosomal hydrolases (19, 48). However, the regulatory role of TBC1D5 toward the retromer remains controversial. Ana et al. reported that loss of retromer or TBC1D5 causes the hyperactivation of Rab7, accompanied by the striking loss of its motility and membrane cycle, but abnormally activated Rab7 has no impact on the recycling of retromer-dependent cargoes under normal conditions (36). Inhibition of TBC1D5 can also enhance the recruitment of the retromer through the activation of Rab7a (37). However, Jia et al. reported that loss of TBC1D5 causes defective trafficking of CI-MPR, possibly by affecting the uncoating of the retromer (38). We speculate that the different results of these studies may be attributed to different cell types and conditions and the degree of the reduction of TBC1D5.

**Figure 6:**
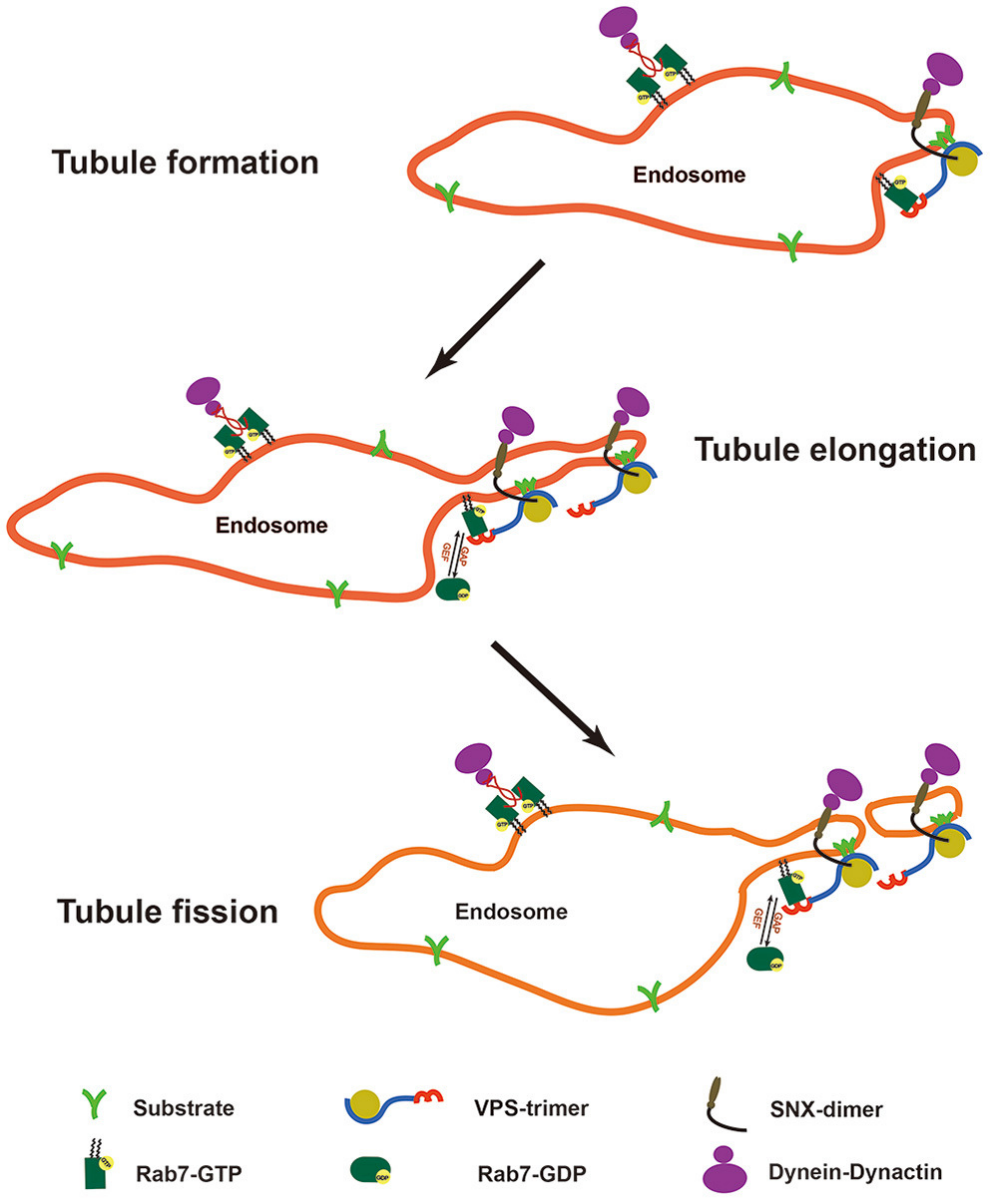
Model of the Rab7 membrane cycle and changes in retromer tubules. Rab7 localizes at the endosomal foci to recruit the retromer-cargo complex, and the regional enrichment of the cargoes contributes to the formation of the retromer tubule. Rab7 gradually accumulates at the endosomal foci to recruit more retromer-cargo complexes and contributes to the elongation of the retromer tubule with the help of the SNX dimer and the cytoskeleton. However, active Rab7 is absent from the tubule structure. Finally, rab7 is inactivated to release the energy source required for the scission of the retromer tubule.

Deficiency of TBC1D5 can result in the hyperactivation of Rab7 and cause defects in its membrane cycle (36). However, how TBC1D5-Rab7 regulates the process of retromer-mediated cargo transport remains unclear. Previous studies have focused on the membrane recruitment function of Rab7 (28, 29). Using a yeast model in which the recruitment of the retromer is independent of Ypt7, Liu et al. found that inhibition of the activation of Ypt7 causes the accumulation of Vps10 on retromer-decorated endosomes, which suggests that Ypt7 also regulates the export of the retromer-cargo complex (49). Another study using live cell imaging revealed changes in the Rab7 signal during retromer-mediated cargo transport. Rab7 localizes at the endosome foci where the retromer tubule emanates, gradually accumulating as the tubule elongates but sharply decreasing during fission of the tubule (28). In the present study, we found that loss of TBC1D5 inhibited the dissociation of the retromer. Combining previous studies and our results, we can deduce that active Rab7 recruits and accumulates the retromer-cargo complex in local endosomes and that Rab7 is inactivated and released from endosomes to promote scission of the retromer tubule. Consequently, the whole process requires the efficient membrane cycle of Rab7 (Figure 6).

However, it is still unclear how the cycle of Rab7 regulates the export of the retromer-cargo complex. In our study, we found that transfection of TBC1D5 siRNAs impeded the binding of the retromer to microtubules and p150^glued^ dynactin. Dynein-dynactin complexes are important microtubule motors that interact with Rab7 through Rab7-interacting lysosomal protein (RILP) to regulate the minus-end transport of endosomes, and Rab7 can also interact with kinesin through FYCO1 to regulate plus-end transport (27, 50–52). However, active Rab7 preferentially retains the activity of dynein compared with kinesin (53). Overexpression of Rab7 causes a decrease in endosome motility, and overexpression of the constantly active form causes an even more severe phenotype (54). We speculate that apart from the increased fusion events with Rab7 overexpression, constantly active Rab7 could occupy the dynein-dynactin complex, which decreases the transient and efficient interaction. Loss of efficient interaction decreases the longitudinal force generated from the microtubule motors, which is indispensable for the formation and stabilization of the retromer tubule and the tubule scission process (20, 44). In addition, a lack of TBC1D5 also decreases ATP generation from Rab7 GTP hydrolysis, which is needed for membrane scission. Consequently, we can conclude that the proper membrane cycle of Rab7 regulated by TBC1D5 is necessary for the detachment of the retromer through affecting the binding of retromer to the microtubule and the p150^glued^ of dynactin.

## Conclusions

In summary, our work uncovered a new mechanism of lysosomal dysfunction in ischemic cardiomyocytes. We found that TBC1D5 deficiency impaired the function of the retromer by affecting its connection to the microtubule, which decelerated the dissociation of the retromer-cargo complex. I/H treatment caused a decrease in TBC1D5 and dysfunction of the retromer, which inhibited the trafficking of lysosomal cathepsins, and restoration of TBC1D5 improved this process. Studies have shown that the promotion of lysosomal function can be protective for the ischemic heart. Our study sheds light on the important role of retrograde transport in the regulation of lysosomal function in ischemic conditions and suggests that TBC1D5 might be a possible therapeutic target for the ischemic heart.

## Data Availability Statement

The original contributions presented in the study are included in the article/Supplementary Material, further inquiries can be directed to the corresponding author/s.

## Ethics Statement

The animal study was reviewed and approved by the Animal Experiment Ethics Committee of the Army Medical University.

## Author Contributions

LC and QZ carried out the experimental work and collected the data. LC drafted the manuscript. YaH and LY participated in the design and coordination of the experimental work. JZ and XJ participated in the study design and the interpretation of the data. JJ and YL participated in the coordination of the experimental work. DZ and YuH designed the study and carried out the data analysis and the modification of the manuscript. All authors contributed to the article and approved the submitted version.

## Funding

This study was supported by the Key Project of National Natural Science Foundation of China (NSFC) (No. 81430042) and the State Key Laboratory of Trauma, Burns, and Combined Injury [No. SKLZZ2012(III) 01].

## Conflict of Interest

The authors declare that the research was conducted in the absence of any commercial or financial relationships that could be construed as a potential conflict of interest.

## Publisher's Note

All claims expressed in this article are solely those of the authors and do not necessarily represent those of their affiliated organizations, or those of the publisher, the editors and the reviewers. Any product that may be evaluated in this article, or claim that may be made by its manufacturer, is not guaranteed or endorsed by the publisher.
