# Supplementary material for: Impaired Retrograde Transport Due to Lack of TBC1D5 Contributes to the Trafficking Defect of Lysosomal Cathepsins in Ischemic/Hypoxic Cardiomyocytes
Source: Front Cardiovasc Med. 2021 Dec 23;8:796254. doi: 10.3389/fcvm.2021.796254 (PMC8736705; doi:10.3389/fcvm.2021.796254)
Supplement: Supplementary file 1 [file Data_Sheet_1.docx]

**Supplementary Figures**

**
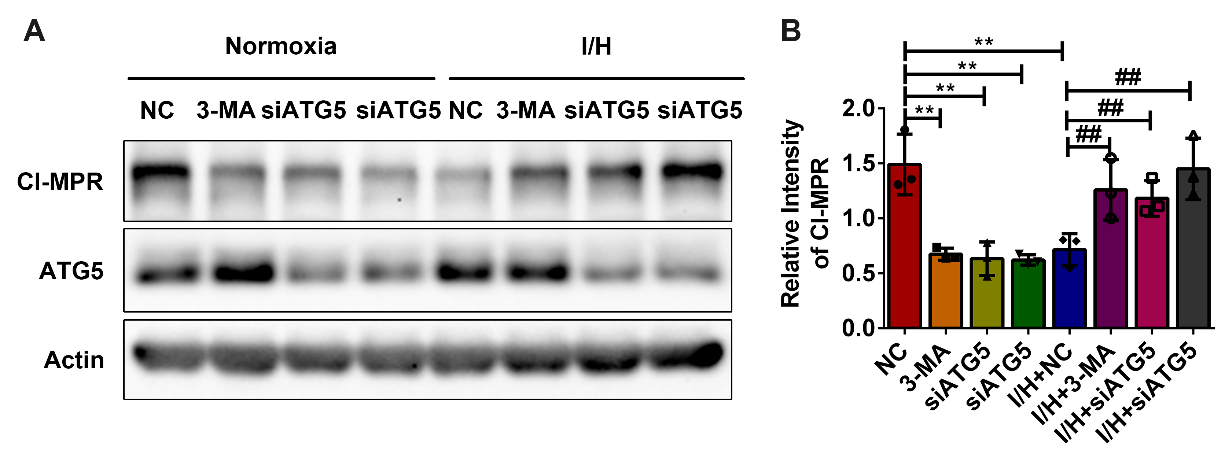
**

**Supplementary Figure S1 Autophagy regulates the protein level of Cl-MPR in cardiomycytes.** (**A**) 3-MA (1mM) and ATG5 siRNAs were applied to inhibit the induction of autophagy in both control and I/H conditions. The protein level of Cl-MPR was detected with western blotting. (**B**) Quantitative analysis of the immunoblots in (**A**). All error bars = SD, **P < 0.01 versus the control cells and ^##^P < 0.01 versus the I/H group.


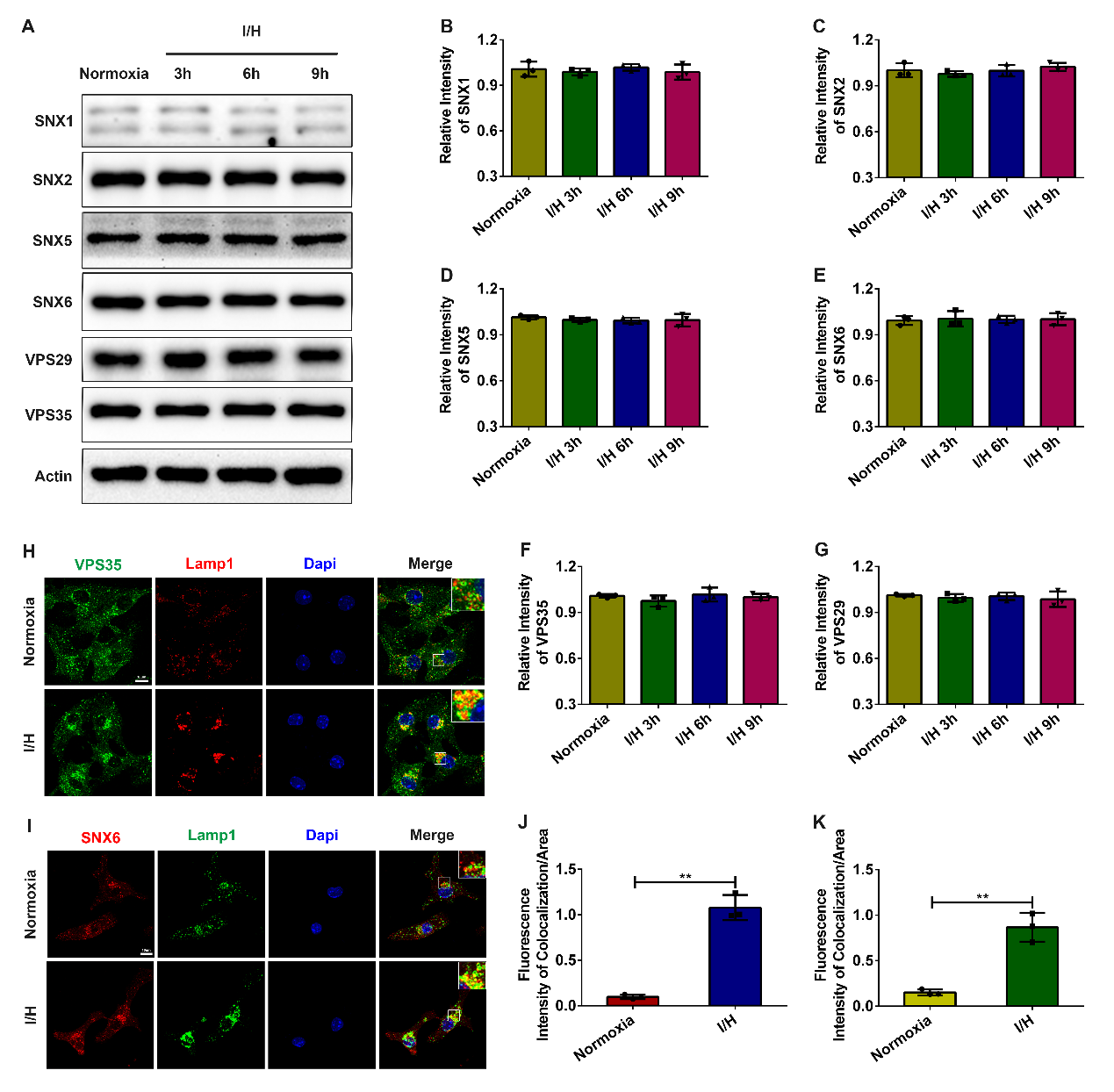


**Supplementary Figure S2 The changes of the retromer complex with I/H treatment in cardiomyocytes.** (**A**) Western blotting was performed to detect the protein levels of the retromer complex with different periods of I/H treatments. (**B-G**) Quantitative analysis of SNX1 (**B**), SNX2 (**C**), SNX5 (**D**), SNX6 (**E**), VPS35 (**F**), VPS29 (**G**) in (**A**). There were no statistic differences among the groups. (**H and J**) The connection of the retromer with the endosomal membrane was analyzed by the [immunofluorescent](javascript:;) colocalization [staining](javascript:;) of VPS35 and Lamp1 (**H**) and the quantitative analysis of the colocalization intensity (**J**). (**I and K**) Representative images of the [immunofluorescent](javascript:;) colocalization [staining](javascript:;) of SNX6 and Lamp1 (**I**) and the quantitative analysis (**K**). All scale bars, 10µm. All error bars = SD, **P < 0.01 of the respective condition compared to the control group.


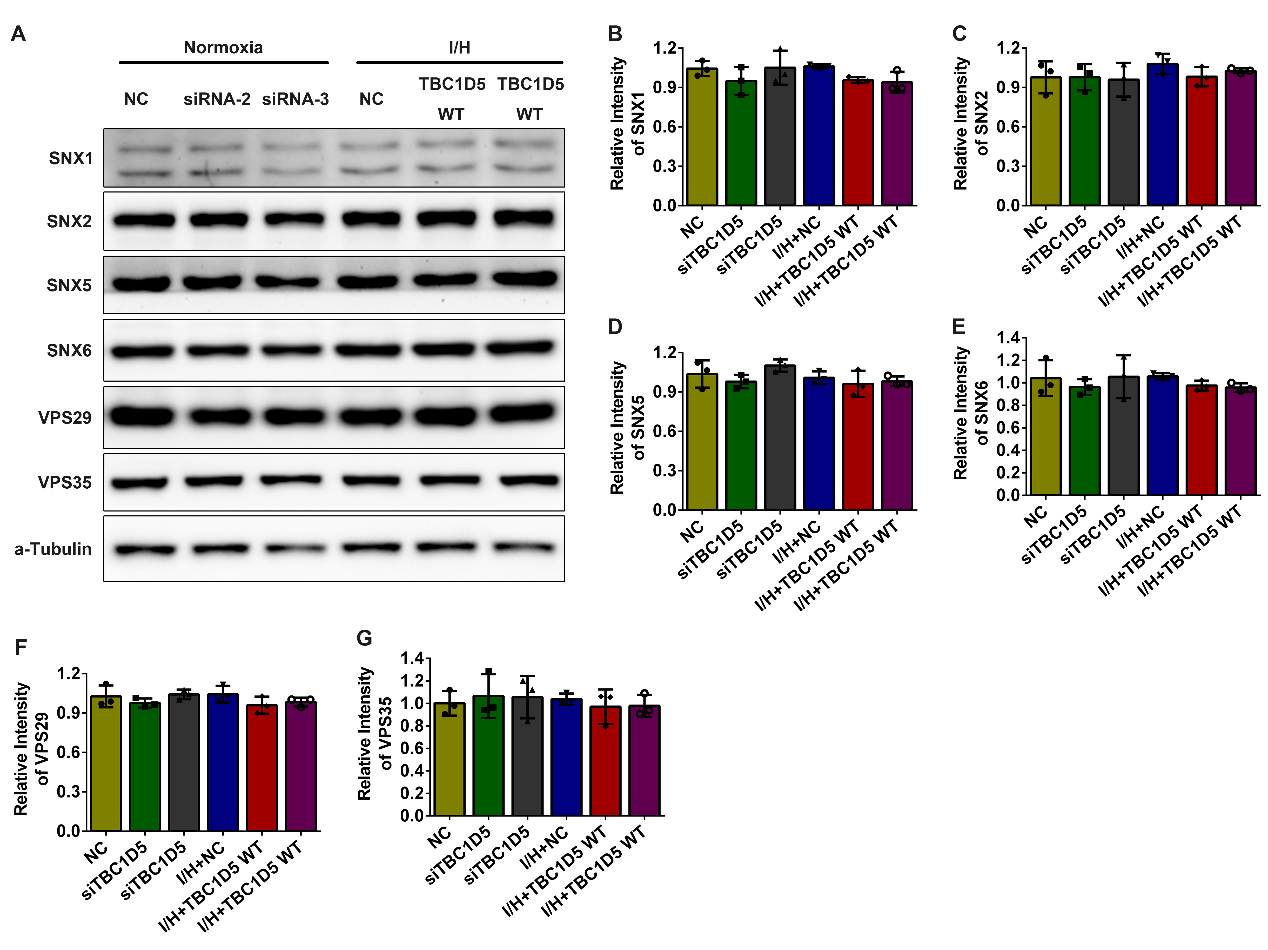


**Supplementary Figure S3 TBC1D5 don’t affect the protein levels of the retromer complex in cardiomyocytes.** (**A**) Western blotting was performed to detect the protein levels of the retromer complex with TBC1D5 siRNAs in normal conditions and TBC1D5 adenovirus in I/H conditions. (**B-G**) Quantitative analysis of SNX1 (**B**), SNX2 (**C**), SNX5 (**D**), SNX6 (**E**), VPS35 (**F**), VPS29 (**G**) in (**A**) and there were no statistic differences among these groups.
